# Origin of the improved mobility and photo-bias stability in a double-channel metal oxide transistor

**DOI:** 10.1038/srep03765

**Published:** 2014-01-20

**Authors:** Hong Yoon Jung, Youngho Kang, Ah Young Hwang, Chang Kyu Lee, Seungwu Han, Dae-Hwan Kim, Jong-Uk Bae, Woo-Sup Shin, Jae Kyeong Jeong

**Affiliations:** 1 Department of Materials Science and Engineering, Inha University, Incheon 402-751, Republic of Korea; 2 Department of Materials Science and Engineering and the Research Institute of Advanced Materials, Seoul National University, Seoul 151-742, Republic of Korea; 3 R&D Center, LG Display Company, Paju-Si, Kyonggi-Do 413-811, Republic of Korea

## Abstract

This study examined the performance and photo-bias stability of double-channel ZnSnO/InZnO (ZTO/IZO) thin-film transistors. The field-effect mobility (μ_FE_) and photo-bias stability of the double-channel device were improved by increasing the thickness of the front IZO film (*t_int_*) compared to the single-ZTO-channel device. A high-mobility (approximately 32.3 cm^2^/Vs) ZTO/IZO transistor with excellent photo-bias stability was obtained from Sn doping of the front IZO layer. First-principles calculations revealed an increase in the formation energy of O vacancy defects in the Sn-doped IZO layer compared to the IZO layer. This observation suggests that the superior photo-bias stability of the double-channel device is due to the effect of Sn doping during thermal annealing. However, these improvements were observed only when *t_int_* was less than the critical thickness. The rationale for this observation is also discussed based on the oxygen vacancy defect model.

Metal oxide thin-film transistors (TFTs) have attracted considerable attention for applications in the active-matrix (AM) backplanes of liquid crystal displays (LCDs) and organic light-emitting diode (OLED) displays due to their high mobility, low production cost, and low-temperature processing capability^1^,^2^,^3^,^4^. Despite these advantages, the high instability of metal oxide TFTs to photo bias is a critical issue that must be resolved to implement such TFTs in applications. Accordingly, the degradation mechanism of negative-bias illumination stress (NBIS)-induced instability has been studied extensively. Three NBIS degradation mechanisms have been suggested, including photo-created hole trapping^5^, photo-ionization of oxygen vacancy defects^6^,^7^, and ambient interactions^8^. The improvement in the NBIS stability of metal oxide TFTs has been achieved based on an understanding of these degradation mechanisms.

High-quality gate insulators, such as SiO_2_ or Al_2_O_3_, which were deposited by plasma-enhanced chemical vapor deposition (PECVD) or atomic layer deposition, were reported to effectively suppress the NBIS instability of the resulting metal oxide TFTs. This suppression was attributed to the large hole injection barrier (>2.0 eV) and low interfacial trap density of these insulators^9^. Dynamic interactions between the oxide channel and ambient gases, such as oxygen or moisture, can be prevented by choosing the appropriate passivation layer on the back channel^10^. The photo-ionization of existing oxygen vacancy (*V_O_*) defects is related to the intrinsic nature of metal oxide semiconductor materials. The incorporation of carrier suppressors, such as Zr, Hf, and Ga, to the relevant metal oxide semiconductor can help reduce the NBIS instability because these suppressors can effectively reduce the concentration of *V_O_* defects in the resulting channel layer. However, the field-effect mobility of the resulting oxide TFTs was adversely affected by the addition of such suppressors^11^,^12^.

The incorporation of In cations to multi-component ZnO-based channels is one of best ways to enhance the field-effect mobility of the resulting oxide TFTs^4^. Unfortunately, increasing the In fraction accelerates the deterioration of NBIS instability of the metal oxide TFTs^13^ due to the higher rate of formation of *V_O_* defects under In-rich conditions^14^. Consequently, the trade-off relationship between mobility and photo-bias stability in a single channel makes it difficult to produce oxide TFTs with both good bias stability and high field-effect mobility. As an alternative approach, a double-channel structure can provide a solution to the adverse tradeoff between field-effect mobility and photo-bias stability.

Several studies have assessed the benefits of a double-channel structure^15^,^16^,^17^,^18^,^19^. Chong et al. reported the insertion effect of Ga-doped ZnO (GZO) at the interface between IGZO and the SiO_2_ gate insulator on the mobility and positive-bias stress (PBS) instability of the resulting oxide TFTs. The improvement of both the mobility and PBS stability of IGZO/GZO TFTs was attributed to the localization effect of the conducting channel path, where the inserted conducting GZO layer reduced the screening length of the channel layer under an on-state current condition while suppressing the trap-induced PBS instability at the channel bulk and back surface region^17^. Kim et al. reported the density-of-states (DOS)-based design of the double-channel device^18^. The optimized HIZO/IZO TFTs exhibited improvement in both the field-effect mobility and NBIS stability compared to those of the single-channel devices. The authors also reported the superior mobility and gate bias stability of ZTO/ITO double-channel devices^19^. Nevertheless, these studies did not provide an in-depth theoretical explanation of the improvements.

In this study, double-channel oxide TFTs consisting of a back-layer ZTO film and front-layer IZO film were fabricated. The thickness-dependent performance and stability of the front IZO layer were examined experimentally. The optimum device was achieved for the 5-nm-thick IZO device, which exhibited a high field-effect mobility (μ_FE_) of 32.3 cm^2^/Vs, a low subthreshold gate swing (SS) of 0.12 V/decade, a reasonable threshold voltage (V_th_) of 0.5 V, a high I_on/off_ ratio of > 10^8^, and the best NBIS stability. A depth profile analysis of the channel stack revealed the inter-diffusion of Sn and In atoms, which occurs during thermal annealing at 500°C. Therefore, the front IZO layer was doped with Sn cations. The microscopic origin for such improvement was examined by first-principles calculations based on density functional theory. The formation energy of oxygen vacancies for the IZO front layer substantially increased (by approximately 0.3 eV) due to Sn doping, which is responsible for the improvement in the NBIS stability of the double-channel device.

## Results

**Figure 1:**
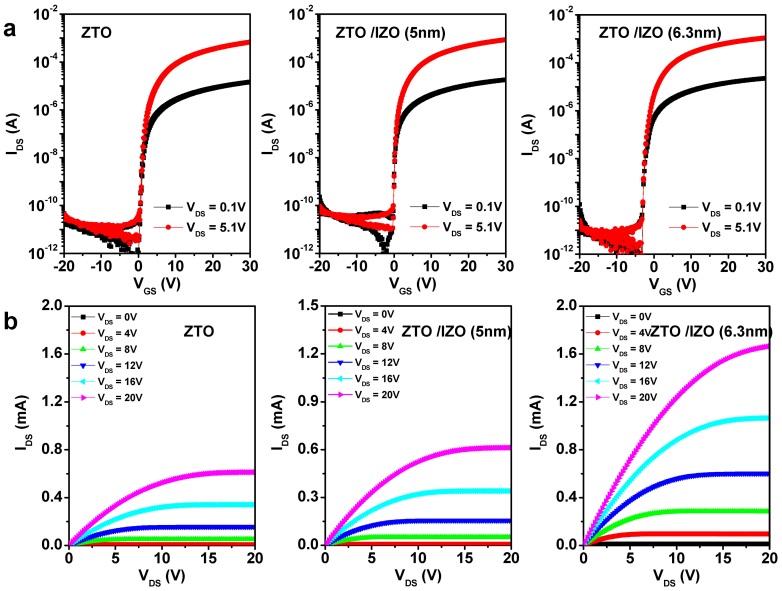
Representative transistor performance of the ZTO control device and ZTO/IZO devices. (a) Transfer characteristics of the ZTO control device, ZTO/IZO (5 nm) device, and ZTO/IZO (6.3 nm) device. The ratio of [O_2_]/([Ar] + [O_2_]) was fixed to 0.3 during deposition of the front IZO layer. (b) The corresponding output characteristics of the ZTO control device, ZTO/IZO (5 nm) device, and ZTO/IZO (6.3 nm) device.

Figure 1a presents the transfer characteristics of the ZTO (control), ZTO/IZO (***t_int_*** = 5.0 nm), and ZTO/IZO (***t_int_*** = 6.3 nm) devices. μ_FE_ was determined by the maximum trans-conductance at a drain voltage (V_DS_) of 0.1 V, and V_th_ was determined from the gate voltage (V_GS_) required to produce a drain current of L/W × 10 nA at V_DS_ = 5.1 V. The subthreshold gate swing (SS = dV_GS_/dlogI_DS_) was extracted from the linear portion of a plot of the log I_DS_ versus V_GS_. For the first-series devices, the front IZO layer was prepared at an oxygen ratio ([O_2_]/[Ar + O_2_]) of 0.3. The single ZTO control device exhibited a μ_FE_, SS, V_th_, and I_on/off_ ratio of 28.8 cm^2^/Vs, 0.20 V/decade, 1.4 V, and 1.5 × 10^8^, respectively. The transporting properties of the double-channel devices were improved substantially by increasing the thickness of the front IZO layer, as summarized in Supplementary Table S1. Therefore, the high μ_FE_ value obtained from the ZTO/IZO (***t_int_*** = 6.3 nm) devices was 33.0 cm^2^/Vs. The enhanced transporting properties of the double-channel device were reflected in the superior output characteristics, as shown in Figure 1b. In contrast, the V_th_ of the double-channel devices were displaced to the negative direction with increasing ***t_int_***. This result indicates that the number of free electron carriers (N_d_) in the channel layer is proportional to the thickness of the front IZO layer. The high mobility of the double-channel devices was attributed to the increasing In content caused by the intercalation of In atoms with a larger radius^1^,^4^. In terms of V_th_, the ZTO/IZO (***t_int_*** = 5.0 nm) device was found to be optimal: a low voltage of 0.41 V is desirable for low-power-consumption AM panels. A similar behavior was observed at a higher oxygen ratio ([O_2_]/[Ar + O_2_]) of 0.4, as listed in Supplementary Table S1.

The SS value of a given TFT device is related to the interfacial trap density (D_it,max_) according to the following equation^20^: 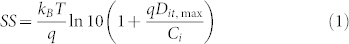
where k_B_ is Boltzmann's constant, T is the absolute temperature, q is the electron charge, and C_i_ is the gate capacitance per unit area. The D_it,max_ of the resulting devices decreased as ***t_int_*** was increased for an oxygen ratio of 0.3 and 0.4, respectively. This increase strongly suggests that the interfacial properties between the SiO_2_ gate insulator and oxide semiconductor channel layer were improved by inserting a front IZO layer. The physical implication will be discussed later in conjunction with the ***t_int_***-dependent bias and photo-bias stability.

**Figure 2:**
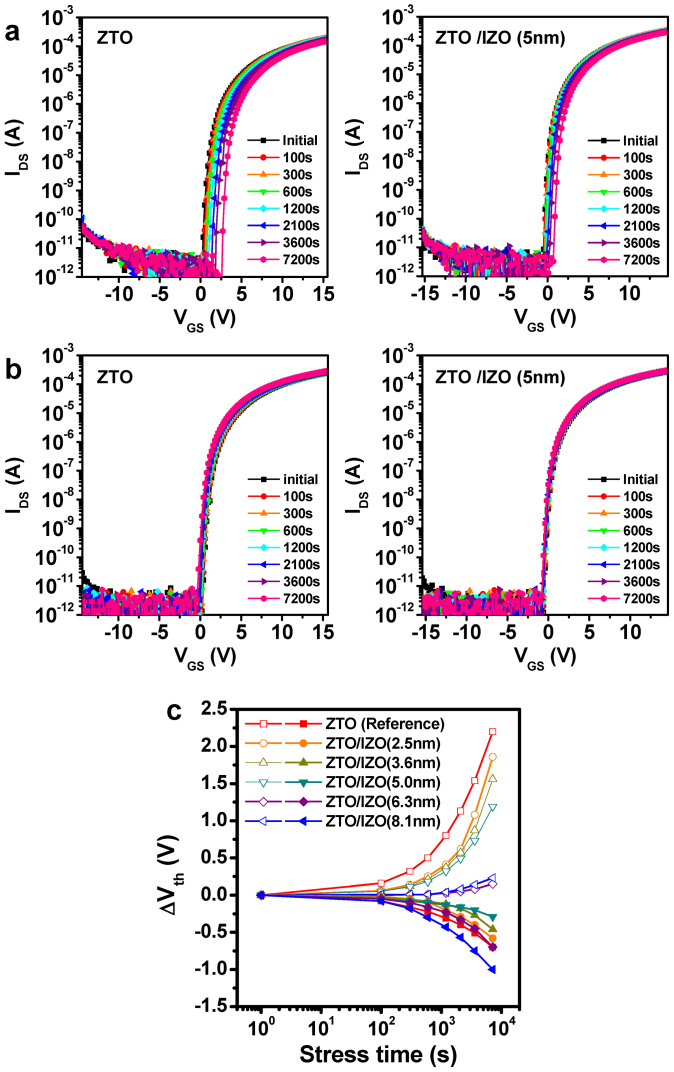
Gate bias stress stability of the ZTO control device and ZTO/IZO device. (a) Evolution of the transfer characteristics of the ZTO control device and ZTO/IZO (5 nm) device with increasing PBS time. (b) The variation of the transfer characteristics for the ZTO control device and ZTO/IZO (5 nm) device as a function of the NBS time. (c) Change in V_th_ for various devices. Empty and filled symbols denote the PBS- and NBS-induced variations, respectively.

The effects of the PBS and negative gate bias stress (NBS) on the transfer characteristics of both devices were investigated. Figure 2a presents the evolution of the transfer curve as a function of the applied PBS time for the control and ZTO/IZO (***t_int_*** = 5.0 nm) devices. The devices were stressed under the following conditions: V_GS_ and V_DS_ were set to 20 V and 5.1 V, respectively, at room temperature, and the stress duration was 7,200 s. For the control device, a parallel V_th_ shift of 2.2 V to a higher voltage with increasing PBS time was observed without an accompanying change in μ_FE_, SS, and the I_on/off_ ratio. The V_th_ stability of the IGZO TFTs under PBS was clearly improved by adopting a double-channel structure (see Fig. 2a). It is noted that a V_th_ shift was further suppressed to ~0.2 V for ZTO/IZO (***t_int_*** = 6.3 and 8.3 nm) devices.

The effect of the channel structure on the NBS instability of the resulting TFTs was also investigated. The devices were stressed under a V_GS_ of −20 V and a stress duration of 7,200 s. Again, the NBS-induced variation was reduced significantly from −0.7 V (control device) to −0.3 V for the double-channel device, as shown in Figure 2b. Figure 2c presents the PBS- and NBS-induced V_th_ variations of the ZTO/IZO devices prepared at an oxygen ratio of 0.3. The PBS-induced V_th_ displacement decreased significantly with increasing ***t_int_*** suggesting that the front IZO film provides a rapid carrier path and lower effective trap density. The V_th_ instability by the application of NBS was also suppressed by increasing ***t_int_***, except for ***t_int_*** ≥ 6.3 nm.

**Figure 3:**
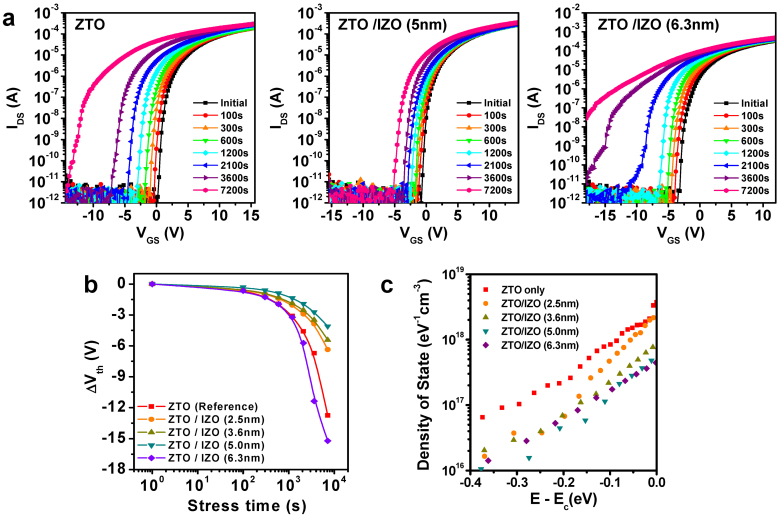
Photo-bias stability of the ZTO control device and ZTO/IZO device. (a) Time evolutions of the transfer characteristics for the ZTO control device, ZTO/IZO (5 nm) device, and ZTO/IZO (6.3 nm) device under NBIS conditions. (b) Variation of V_th_ shift under NBIS conditions for various devices. (c) Calculated DOS distribution as a function of the energy (E – E_C_) for the devices examined.

The photo-bias instability of the ZTO and ZTO/IZO devices was compared, as shown in Figure 3a. A green LED light source with a photo intensity of 0.27 mW/cm^2^ was used under identical NBS conditions. Although the NBIS instability for each device was worse than the dark NBS instability, the changes in V_th_ by the application of NBIS were similar to those by NBS. When ***t_int_*** was less than the critical thickness (***t_crit_***), the NBIS stability of the double-channel device improved with increasing ***t_int_***. Here, *t_crit_* is defined as the maximum thickness that results in higher mobility and better photo-bias stability compared to the single-channel device. For example, the negative V_th_ shift of the double-channel device (***t_int_*** = 5 nm) was reduced from 12.5 V (single ZTO device) to 4.1 V (see Fig. 3a). The double-channel structure (***t_int_*** ≤ ***t_crit_***) exhibited superior photo-bias stability, and the resulting oxide TFTs exhibited improved transporting properties compared to the control device. This result is difficult to understand because higher-In-containing oxide TFTs suffer from severe NBIS degradation due to the easy formation of oxygen vacancy defect centers. However, when ***t_int_*** > ***t_crit_***, the NBIS stability as well as NBS stability of the resulting double-channel device deteriorated abruptly. Therefore, the 6.3-nm-thick device fabricated at an oxygen ratio of 0.3 suffered from the most severe degradation (ΔV_th_ = −15.2 V), which is larger than that of the ZTO control device, as shown in Figures 3a and b.

To obtain more insight into these anomalous behaviors, the DOS distributions of the devices in this study were calculated using the Meyer-Neldel rule^21^. Figure 3c presents the DOS distribution as a function of the energy for the single- and double-channel devices. The overall DOS distribution of the double-channel device (***t_int_*** = 5 nm) was much lower than that of the single-channel device. Here, the extracted DOS distributions are the summation of the bulk trap states (N_SS_) in the channel region and N_it_ at the channel/gate insulator interface. Because the thickness of the back ZTO channel was fixed to 35 nm for all devices, the N_SS_ values for both devices would be comparable. Therefore, the In-rich composition can effectively eliminate the N_it_ at the IZO/gate insulator interface compared to those at the ZTO/gate insulator interface, which is consistent with the N_it,max_ variations extracted from the SS values.

The PBS instability can be understood based on the carrier trapping mechanism. The amount of charge trapped into the channel bulk and at the channel/insulator interface will be proportional to the number of trap states regardless of the charge polarity. Therefore, the improvement in the PBS stability of the double structure devices can be attributed to the effective decrease in the interfacial trap density compared to that of the single-channel device. This inverse proportionality between the gate bias instability and DOS distribution was observed for all ***t_int_*** values. The MNR-derived DOS energy range was limited from the conduction band edge (E_C_) to (E_C_ −0.4 eV), which corresponds to the tailing state of acceptor-like traps. Because the quasi-Fermi energy (E_F_) at the channel interface is positioned near the E_C_ edge under the PBS condition, this DOS distribution directly affects the PBS instability, as discussed earlier. In contrast, NBS and NBIS instability mainly results from the V_O_ deep state. Therefore, a complete understanding of photo-bias instability requires the comparative characterization of the V_O_ density by X-ray photoelectron spectroscopy (XPS) and the theoretical electron calculation of V_O_ defects.

**Figure 4:**
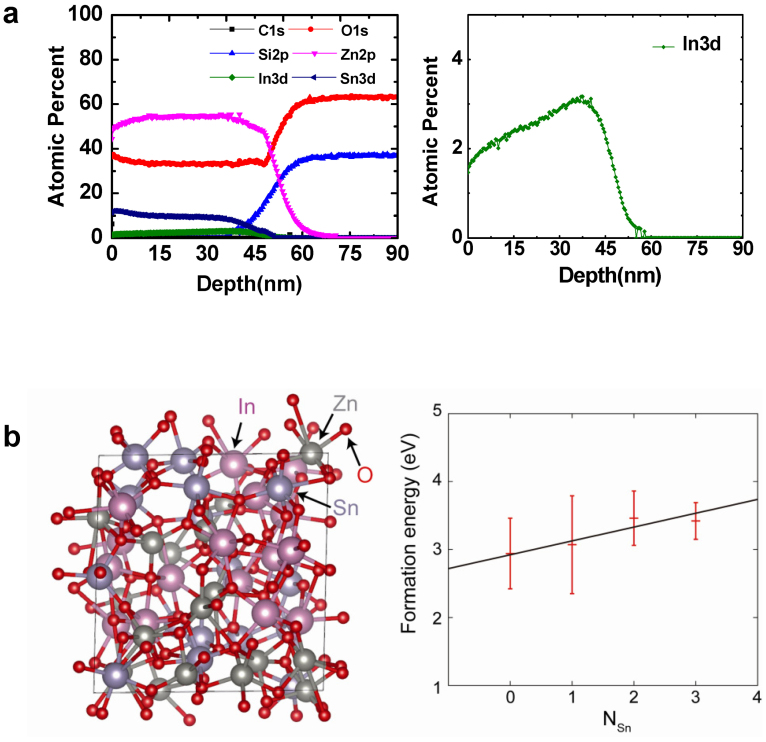
Composition analysis of the ZTO/IZO device and electron calculation results. (a) Depth profile of various elements for the ZTO/IZO(5 nm)/SiO_2_ stack structure. The enlarged depth profile of In is also shown. (b) Amorphous structure of ITZO, including eight formula units of In_2_Sn_2_Zn_2_O_9_ and the calculated formation energies of *V_O_* as a function of the number of neighboring Sn atoms. The mean values and standard deviation are presented in red, and the black solid line represents a linear regression.

Thus, the atomic distribution and chemical state of a ZTO/IZO film were analyzed by XPS. Figure 4a presents the depth profile of the various elements in the ZTO(35 nm)/IZO(5 nm)/SiO_2_ stack. The Sn and In atoms in the channel stack region inter-diffused during thermal annealing for 1 h at 500°C, suggesting that the front IZO layer was doped with Sn cations. Pre-existing *V_O_* defects in the channel layer have a deleterious effect on the NBIS-induced instability^14^. This negative effect was attributed to a photo transition from the neutral oxygen vacancy (*V_O_*) to the double-charged oxygen vacancy defects (*V_O_*^+2^), which results in the delocalized electrons in the conduction band. The influence of Sn on the *V_o_* formation energy was examined by first-principles calculations using the VIENNA *ab initio* simulation package (VASP)^22^ with the similar computational details to those reported in Ref. 23. The projector-augmented wave (PAW) pseudopotentials^24^ with a kinetic energy cutoff of 500 eV and a 2 × 2 × 2 *k*-point mesh were used. For the exchange-correlation energy of electrons, the generalized-gradient approximation (GGA) + *U* calculation was used because it partially corrects the band-gap problem in GGA by decreasing the repulsion between oxygen *p* and metal *d* states^25^. The effective *U* parameters were set to 7.0, 3.5, and 8.0 eV for the In 4 *d*, Sn 4 *d*, and Zn 3 *d* states, respectively.

The amorphous structure of the InSnZnO (*a*-ITZO) compound with a stoichiometry of n_In_:n_Sn_:n_Zn_ = 1:1:1 was modeled by a first-principles molecular dynamics (MD) simulation. The eight formula units of In_2_Sn_2_Zn_2_O_9_ were distributed randomly into a cubic supercell. The structure was heated to 2,000 K for 5 ps, and then, the temperature was reduced rapidly to 300 K at a −300 K/ps quenching speed. The lattice parameters and atomic positions were fully relaxed, resulting in the final amorphous structure shown in Figure 4b.

The formation energy (E_f_) for *V_O_* defects in the neutral state was calculated using the following formula: 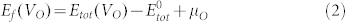
where *E_tot_*(*V_O_*) and 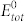 represent the total energy of the supercell with and without *V_O_*, respectively, and μ_O_ is the chemical potential of oxygen under the given growth condition. In the present study, 
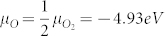
, corresponding to an oxygen-rich limit.

In an amorphous multicomponent oxide, *E_f_*(*V_O_*) depends strongly on the local environment of oxygen^25^. In contrast to the crystal, various types of *V_O_* configurations exist in the amorphous structure. Therefore, 33 different *V_O_* configurations were calculated by removing an oxygen atom in the modeled *a*-ITZO and examining how the neighboring cation species affect the formation energy of the *V_O_*. In Figure 4b, the formation energies were averaged with respect to the neighboring Sn atoms (N_Sn_). The formation energies increase substantially with increasing N_Sn_, indicating that *V_O_* becomes difficult to form as more Sn atoms are introduced to the material. This dependence is consistent with the previous first-principles study that demonstrated the higher formation energy of *V_O_* in crystalline SnO_2_ compared to those of In_2_O_3_ and ZnO^26^.

In addition, a recently proposed model on the peroxide formation through the hole creation inside the valence band may be valid in the present case and may also contribute to the observed photo instability^27^. By forming O-O bonds, the peroxides increase the carrier density in a manner similar to the oxygen vacancy. The tendency for the peroxide formation may increase as the energy gap is reduced. We theoretically observe that the energy gap of IZO is smaller than that for Sn-doped IZO by approximately 0.3 eV. That is, the formation of photo-induced peroxide is also more significant for IZO. Therefore, the improved NBIS stability for the double-channel devices (***t_int_*** < ***t_crit_***) can be attributed to the suppression of *V_O_* defects in the front IZO layer by Sn doping and the reduced N_it_ density at the front layer/gate insulator interface.

**Figure 5:**
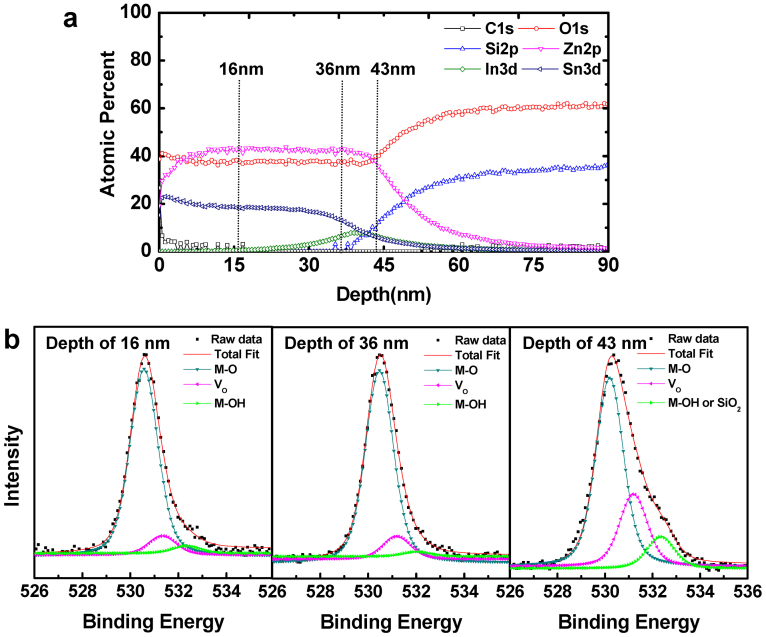
Composition analysis of the ZTO/IZO device. (a) Depth profile of the elements in the ZTO/IZO(8.3 nm)/SiO_2_ stack structure. (b) O1*s* XP spectra of the position at a depth of 16, 36, and 43 nm, which correspond to the bulk ZTO region, near-interfacial ZTO/IZO region, and interfacial IZO/SiO_2_ region, respectively. The O1s spectra were de-convoluted into three different peaks, the “lattice oxygen peak without oxygen vacancies” (530.4 eV), the “lattice oxygen peak in the oxygen deficient region” (531.2 eV), and the “metal hydroxide peak or SiO_2_–related peak” (531.3 eV).

The remaining question is why the thick front channel device (***t_int_*** > ***t_crit_***) suffers from inferior photo-bias stability compared to that of the single-channel device. Figure 5a presents the depth profile of various elements in the ZTO(35 nm)/IZO(8.3 nm) stack at an oxygen ratio of 0.3. The effective diffusion length of Sn atoms was less than the thickness of a front IZO layer. Thus, some portion of the front IZO layer is unaffected by Sn doping during thermal annealing. The existence of an IZO layer with high In content would be responsible for the deterioration of the NBIS stability for a thick front channel device because the formation energy of *V_O_* is reduced due to the weak bonding of In and oxygen.

Figure 5b shows the evolution of the O1s X-ray photoelectron (XP) spectra along the depth direction. The position at a depth of 16, 36, and 43 nm corresponded to the bulk ZTO region, interfacial ZTO/IZO region, and interfacial IZO/SiO_2_ region, respectively. The O1s peaks centered at binding energies of 530.4 ± 0.1 and 531.2 ± 0.2 eV arose from oxygen atoms in the oxide lattices without and from oxygen vacancies, respectively, as summarized in Supplementary Table S2^28^,^29^,^30^. The peak areas of the oxygen vacancies clearly increased with increasing depth. A larger portion of the increased oxygen vacancies at a depth of 43 nm compared to that at the depth of 36 nm suggests that the front IZO layer consists of a Sn-doped and Sn-free IZO layer. Therefore, the double-channel device with a thick front channel device (***t_int_*** > ***t_crit_***) exhibited poorer NBIS stability due to the large *V_O_* concentration of the Sn-free front IZO layer. The extreme case corresponds to the single-IZO-channel device. The oxide TFTs with a 35-nm-thick single IZO channel did not exhibit any I_on/off_ modulation but instead exhibited simple metallic behavior. The N_d_ value of the 35-nm-thick IZO film was determined to be > 10^20^ cm^−3^ from the Hall effect measurement, which is consistent with the thick IZO film (***t_int_*** > ***t_crit_***) with a high *V_O_* concentration.

**Figure 6:**
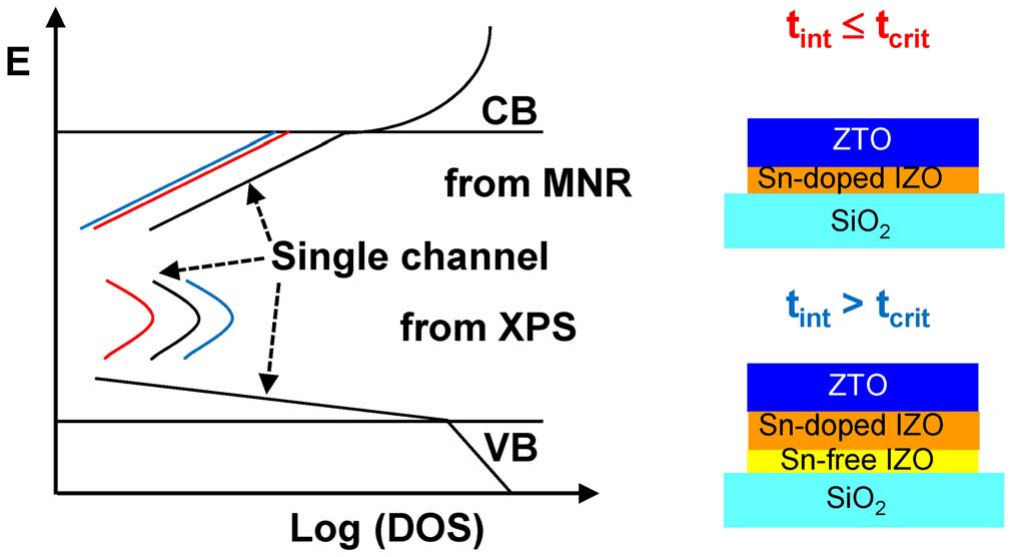
Proposed subgap DOS distribution. The black line denotes the acceptor-like tail state and deep V_O_ states of the ZTO control device. The thin (*t_int_* ≤ *t_crit_*) and thick (*t_int_* > *t_crit_*) IZO devices are represented by the red and blue lines, respectively. The thin IZO double-channel device has fewer interfacial trap states over an entire forbidden bandgap. In addition, the V_O_ formation is strongly suppressed by the Sn-doping effect to the IZO front layer. In contrast, the thick IZO device has extremely deep V_O_ states due to the limited diffusion length of the Sn cation.

Figure 6 provides a schematic comparison of the total subgap DOS distribution for the ZTO control device and the thin (*t_int_* ≤ *t_crit_*) and thick (*t_int_* > *t_crit_*) IZO devices. There are two competing factors governing the PBS, NBS, and NBIS instability of the double-channel devices. One factor is the lower D_it,max_ between the Sn-doped IZO (IZTO) and SiO_2_ insulator compared to that between the ZTO and SiO_2_ insulator. The other factor is the creation of *V_O_* defects. For thin (*t_int_* ≤ *t_crit_*) devices, the suppressed *V_O_* defect and lower D_it,max_ values are responsible for the improvement in the gate bias and photo-bias stability. However, thick (*t_int_* > *t_crit_*) devices suffer from the abrupt creation of *V_O_* in the interfacial In-rich region due to the limited Sn diffusion to the IZO front layer, which leads to the deterioration of the NBS and NBIS stability.

**Figure 7:**
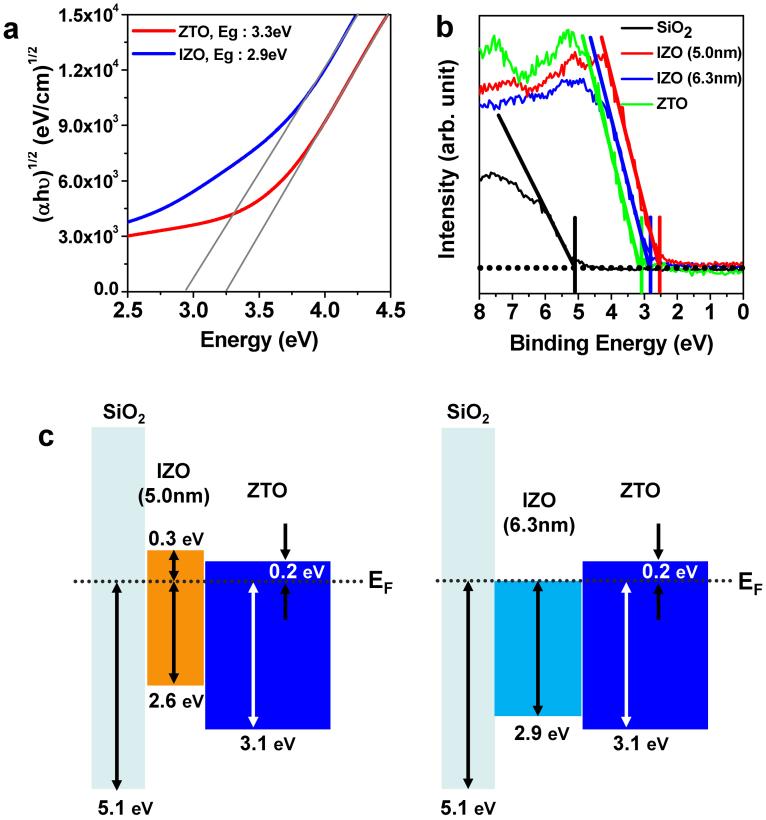
Optical properties and electronic structure of the IZO and ZTO films. (a) Tauc plot; (αhν)^1/2^ as a function of the photon energy, hν, where ν is the photon frequency, indicating the optical bandgap (E_g_) for the IZO (2.9 eV) and ZTO (3.3 eV) films. (b) XP spectra near the VB maximum of the ZTO, 5.0-nm-thick and 6.3-nm-thick IZO, and SiO_2_ films. (c) Proposed energy band diagram of the thin and thick IZO double-channel structures based on the obtained VB profile.

To confirm the proposed origin of the improved mobility and photo-bias stability with the strong t_int_ dependency, the relative band structure for the ZTO/IZO/SiO_2_ stack was further analyzed by XP spectra using a monochromatic Al-K_α_ source. The XP valence band (VB) profiles near the bandgap for the ZTO, 5.0-nm-thick IZO, 6.3-nm-thick IZO, and SiO_2_ are depicted in Figure 7b. The VB maximum of each layer is approximately 3.1, 2.6, 2.9, and 5.1 eV, from the Fermi level for the ZTO, 5.0-nm-thick IZO, 6.3-nm-thick IZO, and SiO_2_, respectively. The band diagrams obtained from the VB spectra and optical bandgaps of the IZO and ZTO films are depicted in Figure 7c. The 5.0-nm-thick IZO film is in a semiconducting state, whereas the 6.3-nm-thick IZO film is in a degenerated conducting state.

This remarkable difference suggests that the thicker IZO film (***t_int_*** > ***t_crit_***) indeed has a high N_d_ due to the high *V_O_* concentration. The ***t_crit_*** value of the double-channel TFTs clearly depends on the content of *V_O_* defect in a front layer. Indeed, the ***t_crit_*** value for the double-channel TFTs increased from 5.0 to 6.5 nm when the oxygen ratio of O_2_/[O_2_ ± Ar] during the sputtering of the IZO layer increased from 0.3 to 0.4, as shown in Supplementary Figure S1. This increase is reasonable because the intentional supply of oxygen species in the IZO front layer slow the abrupt V_O_ formation in the In-rich interfacial region due to the limited diffusion length of the Sn cation. In previous studies, the thickness of the front layer for the optimized double-channel device was in range of 3.5–5.0 nm^18^,^19^. These results suggest that the effective diffusion length of the suppressor cations, such as Hf and Sn, into the In-rich front layer (IZO or ITO) during the given thermal annealing is less than 3.5–5.0 nm.

## Discussion

Double-channel ZTO/IZO TFTs exhibited improved mobility and photo-bias stability compared to single-channel ZTO TFTs when the front IZO thickness was less than the critical thickness. These intriguing properties were partially attributed to the reduced D_it,max_ value of the IZTO/SiO_2_ interface compared to that of the ZTO/SiO_2_ interface for the ZTO control device. The Sn-doping effect on the front IZO layer is also responsible for the superior photo-bias stability of the double-channel devices because the formation energy of *V_O_* defects increased with increasing Sn coordination number. In contrast, the double-channel device with a thick front IZO layer (***t_int_*** > ***t_crit_***) suffered from inferior NBS/NBIS stability and a large negative V_th_ displacement compared to those of the ZTO control device. This poor performance is attributed to the limited diffusion length of the Sn atoms compared to the thickness of the thick front IZO layer during thermal annealing. Therefore, careful control of the ***t_int_*** for the interfacial layer is essential for designing a double-channel device that exhibits improved performance and bias stability compared to the single-channel device. Overall, double-channel ZTO/IZO TFTs can be used as the backplane electronics for high-resolution and large-sized AMOLED and TFT-LCD displays.

## Methods

A 120-nm-thick SiO_2_ thin film as a gate insulator was deposited onto a patterned Mo (200-nm-thick)/glass substrate by PECVD at 380°C. Front IZO thin films with *t_int_* values of 2.5–8.3 nm were deposited onto SiO_2_/Mo/glass substrates by DC sputtering. The working pressure was 0.26 Pa, and the relative oxygen ratio ([O_2_]/[Ar + O_2_]) was varied from 0.30 to 0.40. The DC power density of the InZnO (IZO) target (50 wt% In) was fixed to 2.2 W/cm^2^, whereas the deposition time was varied from 30 to 240 s. A 35-nm-thick ZTO film (back layer) was deposited onto an IZO/Mo/glass substrate. The oxygen flow rate of [O_2_]/[Ar + O_2_] was fixed to 0.10 for all devices. The Zn:Sn composition was approximately 50:50. The active channel was defined using a shadow mask during ZTO/IZO channel formation, and the ITO source/drain (S/D) electrode was deposited using the same sputtering system. The fabricated TFTs had a bottom gate structure with a channel width (W) and length (L) of 1,000 μm and 150 μm, respectively. The devices were annealed in air for 1 h at 500°C. The transfer characteristics of the oxide TFTs were measured using a Keithley 2636 Source Meter at room temperature. The film thickness was determined by ellipsometry and confirmed by X-ray reflectivity measurements. The cation compositions and chemical states of the ZTO and IZO films were examined by X-ray fluorescence spectroscopy (XRF, Themoscientific, ARL Quant'X) and XPS, respectively.

## Author Contributions

H.Y.J., J.-U.B. and W.-S.S. designed this work. J.K.J. and S.H. wrote the manuscript. H.Y.J., A.Y.H. and D.-H.K. performed the experiments and electrical measurements. C.K.L. contributed to the XPS analysis. Y.K. and S.H. performed the theoretical calculations. All authors discussed the results and commented on the manuscript. The project was supervised by J.K.J.

## Supplementary Material

Supplementary Information SI
