# Supplementary material for: Origin of the improved mobility and photo-bias stability in a double-channel metal oxide transistor
Source: Sci Rep. 2014 Jan 20;4:3765. doi: 10.1038/srep03765 (PMC3895876; doi:10.1038/srep03765)
Supplement: Supplementary Information — SI [file srep03765-s1.doc]

**Supplementary Information**

Origin of the improved mobility and photo-bias stability in double-channel metal oxide transistor

Hong Yoon Jung^1^, Youngho Kang^2^, Ah Young Hwang^1^, Chang Kyu Lee^1^, Seungwu Han^*2^, Dae-Hwan Kim^3^, Jong-Uk Bae^3^, Woo-Sup Shin^3^ & Jae Kyeong Jeong^1*^

^1^Department of Materials Science and Engineering, Inha University, Incheon 402-751, Republic of Korea, ^2^Department of Materials Science and Engineering, and Research Institute of Advanced Materials, Seoul National University, Seoul 151-742, Republic of Korea, ^3^R&D Center, LG Display Company, Paju-Si, Kyonggi-Do 413-811, Republic of Korea

Correspondence and requests for materials should be addressed to S.H. ([**hansw@snu.ac.kr**](mailto:hansw@snu.ac.kr)); J.K.J. ([**jkjeong@inha.ac.kr**](mailto:jkjeong@inha.ac.kr))

**ZTO**

**ZTO/IZO (4.7nm)**

**ZTO/IZO (6.5nm)**

**ZTO/IZO (8.3nm)**

**Figure S1.** The representative transfer characteristics of the ZTO and ZTO/IZO double channel oxide TFTs. The front IZO layer was deposited at the higher oxygen ratio (O_2_/[Ar + O_2_]) of 0.4. The critical thickness was determined to be 6.5nm.

**Table S1.** Comparison of the device parameters including *μ_FE_, SS, V_th_, I_on/off_* and *D_it,max_* of the various ZTO/IZO TFTs.

| ***Device ID*** | | | **μ_FE_**  **[cm^2^V^-1^s^-1^]** | ***SS***  **[Vdecade^-1^]** | ***V_th_***  **[V]** | ***I_on_/I_off_*** | ***D_it,max_***  **[eV^-1^cm^-2^]** |
| --- | --- | --- | --- | --- | --- | --- | --- |
| ***[O_2_]/([Ar]+[O_2_])*** | | ***t_int_* (nm)** |
| 0.3  0.3  0.3  0.3  0.3  0.3  0.4  0.4  0.4  0.4 | 0  2.5  3.8  5.0  6.3  8.1  2.9  4.7  6.5  8.3 | | 28.8  29.1  29.8  32.3  33.0  43.5  24.6  31.6  34.4  37.4 | 0.20  0.17  0.16  0.12  0.12  0.14  0.16  0.12  0.09  0.21 | 1.4  0.9  0.7  0.4  -1.7  -3.5  0.3  -0.7  -1.6  -3.2 | 1.5 × 10^8^  9.9 × 10^7^  2.3 × 10^8^  9.0 × 10^7^  3.3 × 10^8^  1.0 × 10^8^  3.3 × 10^8^  4.3 × 10^8^  5.1 × 10^8^  2.3 × 10^8^ | 5.1 × 10^11^  4.0 × 10^11^  3.6 × 10^11^  2.2 × 10^11^  2.2 × 10^11^  2.6 × 10^11^  3.6 × 10^11^  2.2 × 10^11^  1.1 × 10^11^  5.4 × 10^11^ |

**a**

**b**

**Figure S2.** The variations of V_th_ values for the ZTO and ZTO/IZO devices prepared at the oxygen ratio of 0.4 as a function of (a) PBS time and (b) NBS time.

**ZTO**

**ZTO/IZO (6.5nm)**

**Figure S3.** The photo-bias stability of ZTO and ZTO/IZO (6.5nm) devices. The front IZO layer was prepared at the higher oxygen ratio of 0.4. It can be clearly seen that the critical thickness was increased from 5.0 nm (at the oxygen ration of 0.3) to 6.5 nm.

**Figure S4.** Variation of V_th_ shift for the ZTO and ZTO/IZO devices prepared at the oxygen ratio of 0.4 under NBIS conditions.

**Table S2.** Depth profile time-dependent XPS O1*s* result for the annealed ZTO/ITO(8.3nm)/SiO_2_ structure.

| **Depth (nm)** | **O 1*s* peak [eV]** | | |
| --- | --- | --- | --- |
|  | **Lattice oxygen [O_O_]**  (530.4 ± 0.1) | **Oxygen deficient [V_O_]**  (531.2 ± 0.2) | **Hydroxyl or SiO_2_**  (531.3 ± 0.2) |
| 16 | 0.88 | 0.09 | 0.03 |
| 36 | 0.81 | 0.14 | 0.05 |
| 43 | 0.64 | 0.25 | 0.11 |
